# Efficacy and safety of BCMA-directed CAR T-cell therapy in extramedullary relapsed or refractory multiple myeloma: a meta-analysis

**DOI:** 10.3389/fimmu.2026.1792558

**Published:** 2026-04-22

**Authors:** Yan-ling Li, Meng-jiao Li, Jia-jia Li, Dan Chen, Cai-yang Li, Ling Qiu, Ying He, Jian-chun Fan, Shi-hui Ren, Hao Yao, Fang-yi Fan

**Affiliations:** 1 Department of Clinical Medicine, North Sichuan Medical College, Nanchong, Sichuan, China; 2 Department of Hematology, Chinese People’s Liberation Army The General Hospital of Western Theater Command, Chengdu, Sichuan, China; 3 Institute of Basic Medicine, North Sichuan Medical College, Nanchong, Sichuan, China; 4 Institute of Oncology, The First Affiliated Hospital of Hebei North University, Zhangjiakou, Hebei, China; 5 College of Medicine, Southwest Jiaotong University, Chengdu, Sichuan, China; 6 Reproductive Medicine Center, Chinese People’s Liberation Army The General Hospital of Western Theater Command, Chengdu, Sichuan, China; 7 Tissue Stress Injury and Functional Repair Key Laboratory of Sichuan Province, Chengdu, Sichuan, China

**Keywords:** BCMA, CAR T-cell therapy, cytokine release syndrome (CRS), extramedullary disease, immune effector cell associated neurotoxicity syndrome, meta - analysis, relapsed/refractory multiple myeloma

## Abstract

**Background:**

This meta-analysis systematically evaluated the efficacy and safety of B-cell maturation antigen (BCMA)–directed chimeric antigen receptor (CAR) T-cell therapy in patients with relapsed or refractory multiple myeloma (RRMM) with extramedullary disease (EMD).

**Methods:**

PubMed, Embase, Web of Science, and the Cochrane Library were searched for studies published up to December 2024 reporting CAR T-cell therapy in RRMM patients with EMD. Studies were screened according to predefined inclusion and exclusion criteria. Data were extracted and the methodological quality was assessed using the Newcastle–Ottawa Scale (NOS) and the MINORS tool; one low-quality study was excluded. A total of 42 studies were included, comprising 242 RRMM patients with EMD and 1,485 without EMD. Fixed- or random-effects models were applied to pool effect sizes. Primary outcomes included objective response rate (ORR), complete response (CR), progression-free survival (PFS), and overall survival (OS). Secondary outcomes included cytokine release syndrome (CRS) and immune effector cell–associated neurotoxicity syndrome (ICANS).

**Results:**

The pooled ORR and CR rates were 79% (95%CI: 71%–86%) and 42% (95%CI: 32%–51%) in the EMD group, and 90% (95%CI: 86%–93%) and 49% (95%CI: 40%–58%) in the non-EMD group, respectively. Reported median PFS ranged from 3 to 18.8 months in the EMD grouPand from 1 to 38 months in the non-EMD group, while median OS ranged from 6 to 13.9 months and from 12.2 to 38 months, respectively. The pooled incidences of grade ≥3 CRS and ICANS were 18% (95%CI: 8%–27%) and 5% (95%CI: 3%–7%) in the EMD group, compared with 13% (95%CI: 7%–19%) and 6% (95%CI: 2%–9%) in the non-EMD group; none of the differences were statistically significant (P> 0.05). Due to inconsistent reporting and lack of individual patient-level data, hazard ratios and pooled time-to-event analyses were not feasible.

**Conclusion:**

Although RRMM patients with EMD exhibited lower ORR and CR rates than those without EMD, BCMA-directed CAR T therapy demonstrated notable clinical activity with a manageable safety profile. However, no direct comparisons with conventional therapies were performed in this analysis.

**Systematic Review Registration:**

https://www.crd.york.ac.uk/prospero/display_record.php?ID=CRD42025613422, identifier CRD42025613422.

## Highlights

- BCMA-directed CAR T-cell therapy demonstrated substantial clinical activity in RRMM patients with extramedullary disease (EMD), achieving pooled ORR and CR rates of 79% and 42%, respectively.
- Compared with non-EMD patients, those with EMD showed lower response rates and shorter ranges of estimated PFS and OS, suggesting an adverse prognostic impact of extramedullary involvement.
- The incidences of grade ≥3 CRS and ICANS were similar between EMD and non-EMD cohorts, indicating a generally manageable and comparable safety profile across groups.
- Subgroup analyses suggested an association between CAR T construct type and variability in treatment response in treatment response, underscoring the need for optimized CAR designs and prospective studies in this high-risk population.

## 1 Introduction

Multiple myeloma (MM) is a hematologic malignancy characterized by the clonal proliferation of plasma cells within the bone marrow. In this disease, excessive secretion of monoclonal immunoglobulins by malignant plasma cells is frequently observed, leading to multisystem involvement. Osteolytic bone lesions, renal impairment, anemia, and hypercalcemia are commonly reported as its clinical manifestations (1, 2). Despite substantial improvements in survival with novel therapeutic approaches—including proteasome inhibitors, monoclonal antibodies, immunomodulatory agents, autologous stem-cell transplantation, and CAR T-cell therapy—relapse and treatment resistance remain common due to disease’s biological heterogeneity (3–9).

Myeloma cells are usually confined to the bone marrow; however, in a subset of patients, these malignant cells have been shown to disseminate beyond the marrow microenvironment and infiltrate extramedullary organs such as the central nervous system, liver, pleura, lymphatic system, and skin (7, 10, 11). When such extramedullary involvement occurs, extramedullary disease (EMD) is diagnosed—a rare yet highly aggressive clinical phenotype associated with organ dysfunction and markedly poor prognosis. Reported incidences of EMD range from approximately 10% to 30% at initial diagnosis and exceed 30% among relapsed cases (10–14). Although the mechanisms underlying EMD remain incompletely understood, aberrant expression of cell-adhesion molecules (including very late antigen-4(VLA-4), CD44, and P-selectin), dysregulation of CXCR4 signaling, and a high prevalence of RAS pathway mutations have been implicated in its pathogenesis (10, 12).

## 2 Methods

### 2.1 Data sources and literature search

This systematic review and meta-analysis was conducted and reported in accordance with the Preferred Reporting Items for Systematic Reviews and Meta-Analyses (PRISMA) 2020 statement. The study protocol was prospectively registered in the International Prospective Register of Systematic Reviews (PROSPERO; registration number: CRD42025613422).

Two reviewers independently searched PubMed, Embase, Web of Science, the Cochrane Library, and major English-language clinical trial registries to identify studies published up to December 2024 that reported the use of CAR T-cell immunotherapy in patients with relapsed or refractory MM, with or without EMD. A combination of Medical Subject Headings and free-text terms was used, with key search terms including “BCMA CAR T-cell therapy,” “extramedullary disease,” “relapsed/refractory,” and “multiple myeloma,” along with their synonyms and related variants. Studies were screened according to predefined inclusion and exclusion criteria. Relevant data were extracted from eligible studies, and methodological quality was assessed using the Newcastle–Ottawa Scale (NOS) and the Methodological Index for Non-Randomized Studies (MINORS). All statistical analyses and graphical visualizations were performed using R Studio and Stata version 14.0.

### 2.2 Quality assessment

The methodological quality of non-randomized studies was assessed using the MINORS tool, whereas cohort studies were evaluated using the Newcastle–Ottawa Scale (NOS).

### 2.3 Data extraction and risk of bias assessment

Two reviewers independently extracted data from all eligible studies, and any discrepancies were resolved through discussion. Data extraction followed established guidelines for meta-analyses and was performed using a predesigned data collection form. Extracted variables included the first author, year of publication, sample size, intervention details (type of CAR T-cell product), median patient age, number of prior treatment lines, history of autologous stem-cell transplantation, ISS stage, and high-risk cytogenetic abnormalities. Efficacy outcomes included complete response (CR), objective response rate (ORR). Safety outcomes included cytokine release syndrome (CRS) and immune effector cell–associated neurotoxicity syndrome (ICANS). Two reviewers independently assessed risk of bias using MINORS for prospective studies and the Newcastle–Ottawa Scale (NOS) for retrospective studies.

Due to the lack of individual patient-level time-to-event data and heterogeneity in survival reporting across studies, survival outcomes (PFS and OS) were not pooled using formal time-to-event meta-analysis methods, but were instead summarized descriptively based on the reported data.

## 3 Results

### 3.1 Literature search

**Figure 1:**
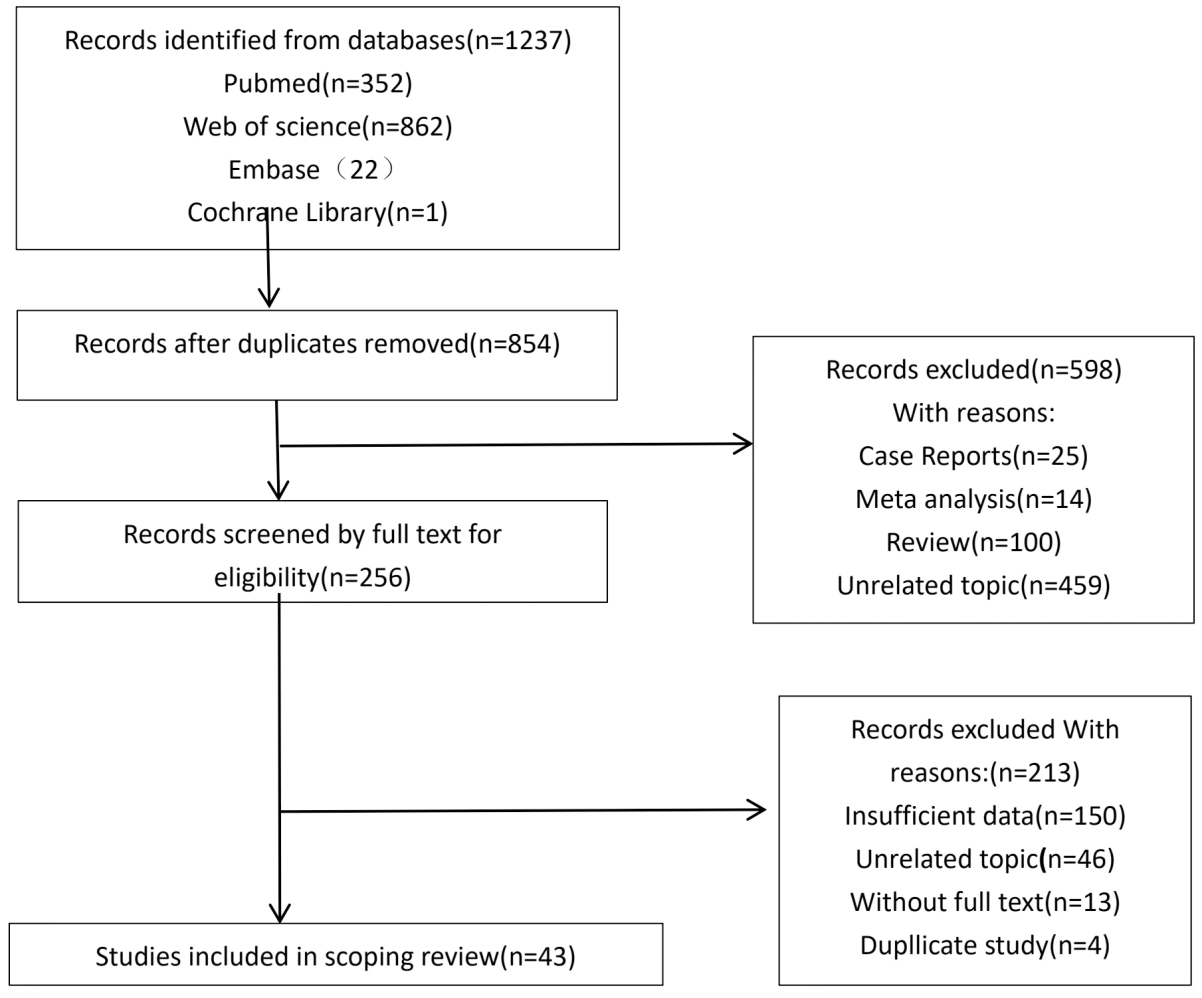
PRISMA flow diagram of study selection. The figure illustrates the literature screening and selection process for the meta-analysis. A total of 1,237 records were identified from PubMed, Embase, Web of Science, and the Cochrane Library. After removal of duplicates, 854 records remained for screening. Based on title and abstract assessment, 598 records were excluded due to being case reports, meta-analyses, reviews, or unrelated topics. The full texts of 256 articles were assessed for eligibility, among which 213 were excluded owing to insufficient data, unrelated topic, lack of full text, or duplication. Ultimately, 43 studies met the inclusion criteria and were included in the qualitative synthesis.

A total of 1,237 studies were initially retrieved from the databases. After removing 383 duplicates, 598 records were excluded during title and abstract screening due to inappropriate publication type or irrelevance to the research topic. The remaining 256 articles underwent full-text assessment, and 213 were excluded because of insufficient data, lack of relevance, unavailability of the full text, or duplicate reporting. One additional study was excluded after quality appraisal owing to a low methodological score. Ultimately, 42 studies were included (15–56), comprising 242 RRMM patients with EMD and 1,485 RRMM patients without EMD (**Figure 1**).

Across these 43 studies, the efficacy and safety of CAR T-cell therapy in RRMM—both with and without EMD—were systematically evaluated. Median patient age ranged across the included studies ranged from 55 to 63 years, with reported age ranges spanning 27 to 81 years. Key baseline characteristics, including sex distribution, median age, prior lines of therapy, CAR scFv origin (murine or humanized), history of autologous stem-cell transplantation, high-risk cytogenetics, and ISS stage, are summarized in **Table 1** (**Table 1A**: EMD cohort; **Table 1B**: non-EMD cohort).

**Table 1A:** Baseline characteristics of the relapsed/refractory multiple myeloma patients with extramedullary disease.

Study	Year	Sample size	Male gende r,n	Median age,years (range)	Median prior lines of therapy (range)	Origin of CAR scFv	Prior autologous SCT, n (%)	High risk cytogenetics, n (%)	ISS stage, n (%)		
									I	II	III
Yimei Que	2021	25	13	55(34-70)	4(3-11)	murine/humanized	–	6(24.0%)	7(30.0%)	11(48.0%)	5(22.0%)
Yiyun Wang	2022	4	3	57(53-69)	4.5(2-7)	humanized	1(25.0%)	–	1(25.0%)	2(50.0%)	1(25.0%)
Baiyan Wang	2020	17	–	55(27-72)	3(1-6)	humanized	–	–	–	–	–
Yuekun Qi	2024	31	17	55(31-70)	4(3-12)	–	18(58.1%)	22(71.0%)	7(33.6%)	9(29.0%)	15(48.4%)
Wei Li	2021	12	4	56(38-69)	11.5(7-17)	murine/humanized	9(75.0%)	10(83.3%)	0	5(41.7%)	7(58.3%)
Haobin Deng	2021	7	4	58(38-73)	12(7-30)	–	2(28.6%)	3(42.9%)	0	4(57.1%)	3(42.9%)
Min Yang	2022	10	6	63(39-70)	4(2-8)	humanized	–	8(80%)	–	–	2 (20.0%)
Danai Dima	2023	64	–	62(43-78)	–	murine/humanized	53 (83.0%)	19(30%)	–	–	–
Wanyan Ouyang	2024	4	–	–	7(6-7)	–	–	3(75.0%)	0	3(75.0%)	1(25.0%)
Siguo Hao	2020	10	–	60(39-70)	4.5(2-11)	humanized	10(41.7%)	–	–	–	9(37.5%)
Min Yu	2024	11	7	55(43-71)	4(2-6)	humanized	2(18.2%)	8(72.7%)	2(18.2%)	2(18.2%)	7(63.6%)
Danai Dima	2024	47	26	60(43-78)	6(4-15)	murine	40(85.0%)	13(35.0%)	5(18.0%)	11(39.0%)	12(43.0%)

**Table 1B:** Baseline characteristics of the relapsed/refractory multiple myeloma patients without extramedullary disease.

Study	Year	Sample size	Male gender, n	Median age, years (range)	Median prior lines of therapy (range)	Origin of CAR scFv	Prior autologous SCT, n (%)	High risk cytogenetics, n (%)	ISS stage, n (%)		
									I	II	III
Shao-long HE	2021	59	35	55(34-70)	4(3-11)	murine/humanized	-	45(76.3%)	24(40.7%)	21(35.6%)	14(23.7%)
Yimei Que	2021	36	23	53(34-69)	4(3-10)	murine/humanized	–	18(50.0%)	16(44.4%)	10(27.8%)	10(27.8%)
Nina Shah	2021	128	–	–	–	murine	–	–	–	–	–
Luise Fischer	2024	27	16	62.15	7(3-13)	murine	–	–	1(7.1%)	7(50.0%)	6(42.9%)
Masaki Ri Saad Zafar	2022	9	5	57(45-71)	5(3-7)	humanized	8(88.9%)	5(55.6%)	5(55.6%)	3(33.3%)	1(11.1%)
Usmani	2021	97	–	–	6(4-14)	humanized	–	–	–	–	–
Kevin R Reyes Sham	2022	78	–	64.5	7(1-14)	murine/humanized	–	–	–	–	–
Mailankody Vladimir	2018	11	–	–	6(4-14)	humanized	11(100%)	9(82.0%)	–	–	–
Vainstein Luciano J.	2023	5	5	64(52-72)	7(4-10)	–	5(100%)	–	3(60.0%)	2(40.0)%	0
Costa	2021	69	39	62.6	5.9	humanized	60(87.0%)	17(25.0%)	–	–	12(17.0%)
Mi Shao Lekha	2021	37	26	61(41-75)	5(2-10)	humanized	8(21.6%)	–	2(18.2%)	2(18.2%)	7(63.6%)
Mikkilineni	2020	21	–	64(41-72)	6(3-12)	humanized	–	9(42.9%)	–	–	–
Jesus G Berdeja	2017	18	–	58(37-74)	7(3-14)	murine	–	–	–	–	–
Rahul Banerjee	2021	50	27	–	–	murine	–	13(26.0%)	–	–	–
Wei Li Andrw J.	2021	9	3	63(42-77)	10(5-16)	murine/human -ized	2(22.2%)	7(77.8%)	0	4(44.4%)	5(55.6%)
Cowan	2019	8	–	64.5(50-70)	10(4-23)	–	–	–	–	–	–
Haobin Deng	2021	13	5	58(42-77)	7(5-16)	–	2(15.4%)	6(46.2%)	1(7.7%)	6(46.2%)	6(46.2%)
Swetha Kambhampati	2022	55	27	62(33-77)	6(1-13)	murine/human -ized	48(87.0%)	–	–	–	–
Nina Shah	2018	8	–	–	9(4-17)	murine	7(87.5%)	4(50.0%)	–	–	–
Bai-Yan Wang	2019	57	34	54(27-72)	4(3-6)	humanized	16(28.1%)	40(70.2%)	10(17.5%)	14(24.6%)	21(36.8%)
Min Yang	2022	14	7	58(39-67)	6(2-11)	humanized	–	4(29.0%)	–	–	7(50.0%)
Danai Dima Wanyan	2023	68	–	67(44-81)	–	murine/human -ized	50(73.53%)	23(33.82%)	–	–	–
Ouyang Robert F	2024	3	–	–	4(3-6)	–	–	1(33.3%)	1(33.3%)	1(33.3%)	1(33.3%)
Cornell	2021	17	–	56	5.5(3-8)	humanized	16(94.0%)	2(12.0%)	–	–	4(36.4%)
Jian-Qing Mi	2022	48	–	61(30-72)	4(3-9)	–	–	–	–	–	–
Deepu Madduri Luciano J.	2019	25	–	61(50-75)	5(3-16)	humanized	–	–	–	–	–
Costa	2022	55	–	62.5(43-75)	5(3-13)	humanized	–	–	–	–	–
Eyal Lebel	2023	50	–	–	5(3-13)	–	–	–	–	–	–
Sarvarinder Kaur Gill Lekha	2023	7	5	65.7(55.1- 77.1)	6(4-10)	murine/human ized	7(100%)	5(71.4%)	–	–	–
Mikkilineni	2019	12	–	63(52-70)	6(3-10)	humanized	–	7(58.3%)	–	–	–
Jennifer Brudno	2017	13	–	54(43-66)	11(3-17)	murine	–	–	–	–	–
Siguo Hao	2020	14	–	60.1(38.5- 69.9)	4.5(2-11)	humanized	10(41.7%)	–	–	–	9(37.5%)
Noopur S. Raje	2021	72	–	–	6(3-17)	murine	–	–	–	–	–
Lijuan Chen Jesus G.	2019	17	–	–	3	–	–	–	–	–	–
Berdeja	2019	22	–	63(42-74)	7(4-17)	murine	18(81.8%)	7(31.8%)	–	–	–
Thomas Martin	2021	97	57	61(43-78)	6(3-18)	humanized	–	–	–	–	–
Melissa Alsina	2020	46	–	62(33-74)	6(3-17)	murine	–	–	–	–	–
Min Yu	2024	9	5	66(54-73)	3(2-4)	humanized	0	7(77.8%)	0	2(22.2%)	7(77.8%)
Danai Dima	2024	105	56	65(41-81)	6(4-15)	murine	77(73%)	36(43%)	10(14.5%)	38(55.1%)	21(30.4%)

### 3.2 Quality assessment

The quality of the included studies was assessed using either the MINORS scale (**Table 2A**) or the Newcastle–Ottawa Scale (NOS) (**Table 2B**), as presented in **Table 2**. The MINORS criteria for non-randomized studies evaluated whether the study objectives were clearly stated, whether patients were consecutively included, whether the anticipated data were collected prospectively, whether the endpoints appropriately reflected the study aims, whether the assessment of endpoints was objective, whether the follow-up duration was adequate, whether the loss to follow-up was less than 5%, and whether a sample size calculation had been performed. The NOS criteria for cohort studies assessed the representativeness of the exposed cohort, the method of selecting the non-exposed cohort, the ascertainment of exposure, and confirmation that the outcome of interest was not present at baseline; the comparability of cohorts based on study design or analytical methods; as well as the adequacy of outcome assessment, the sufficiency of follow-up duration, and the quality and completeness of follow-up.

**Table 2A:** Methodological index for included non-randomized studies.

Study	I	II	III	IV	V	VI	VII	VIII	Total
Danai Dima 2024	2	2	2	2	0	2	2	0	12
Nina Shah 2021	2	2	2	2	0	2	0	0	10
Saad Zafar Usmani 2021	2	2	2	2	0	2	1	0	11
Lekha Mikkilineni 2020	2	2	2	2	0	2	2	0	12
Jesus G Berdeja 2017	2	2	2	2	0	1	0	0	9
Andrw J. Cowan 2029	2	1	1	2	0	1	1	0	8
Nina Shah 2018	2	1	2	2	0	1	1	0	9
Bai-Yan Wang 2019	2	1	2	2	0	2	2	0	11
Min Yang 2022	2	2	2	2	0	2	1	0	11
Wanyan Ouyang 2024	2	1	2	2	0	2	1	0	10
Robert F Cornell 2021	2	2	2	2	0	2	1	0	11
Jian-Qing Mi 2022	2	1	2	2	0	2	1	0	10
Deepu Madduri 2019	2	1	2	2	0	1	0	0	8
Luciano J. Costa 2022	2	1	2	2	0	1	1	0	9
Eyal Lebel 2023	2	1	1	2	0	2	1	0	9
Lekha Mikkilineni 2019	2	1	2	2	0	0	0	0	7
Jennifer Brudno 2017	2	1	2	2	0	0	0	0	7
Siguo Hao 2020	2	1	2	2	0	2	1	0	10
Noopur S. Raje 2021	2	1	2	2	0	2	0	0	9
Lijuan Chen 2019	2	1	2	2	0	2	1	0	10
Jesus G. Berdeja 2019	2	1	2	2	0	1	1	0	9
Thomas Martin 2021	2	1	2	2	0	2	1	0	10
Melissa Alsina 2020	2	1	2	2	0	2	1	0	10

Numbers I–VIII in the heading refer to: I, a clearly stated aim; II, inclusion of consecutive patients; III, prospective data collection; IV, endpoints appropriate for the aim of the study; V, unbiased assessment of the study endpoint; VI, follow-up period appropriate for the aim of the study; VII, loss of follow-up less than 5%; VIII, prospective calculation of the study size.

**Table 2B:** Newcastle-Ottawa quality assessment scale cohort studies.

Study	Selection	Comparability	Outcome	Total
Shao-long HE 2021	3	0	3	6
Yimei Que 2021	4	2	3	9
Yiyun Wang 2022	3	0	3	6
Luise Fischer 2024	3	0	2	5
Baiyan Wang 2020	4	2	3	9
Masaki Ri 2022	3	0	3	6
Yuekun Qi 2024	3	0	3	6
Kevin R Reyes 2022	3	0	3	6
Sham Mailankody 2018	3	0	3	6
Vladimir Vainstein 2023	3	0	2	5
Luciano J. Costa 2021	3	1	2	6
Mi Shao 2021	3	0	2	5
Rahul Banerjee 2021	4	1	3	8
Wei Li 2021	4	1	3	8
Haobin Deng 2021	4	1	3	8
Jessica S.Little 2021	3	0	2	5
Swetha Kambhampati 2022	3	0	2	5
Danai Dima 2023	4	1	3	8
Sarvarinder Kaur Gill 2023	3	0	2	5
Min Yu 2024	2	0	3	5

Selection: 1) Representativeness of the exposed cohort; 2) Selection of the non exposed cohort; 3) Ascertainment of exposure;4) Demonstration that outcome of interest was not present at start of study). Comparability: 1) Comparability of cohorts on the basis of the design or analysis. Outcome: 1) Assessment of outcome; 2) Was follow-up long enough for outcomes to occur; 3) Adequacy of follow uPof cohorts.

### 3.3 Efficacy

#### 3.3.1 Tumor response

**Figure 2:**
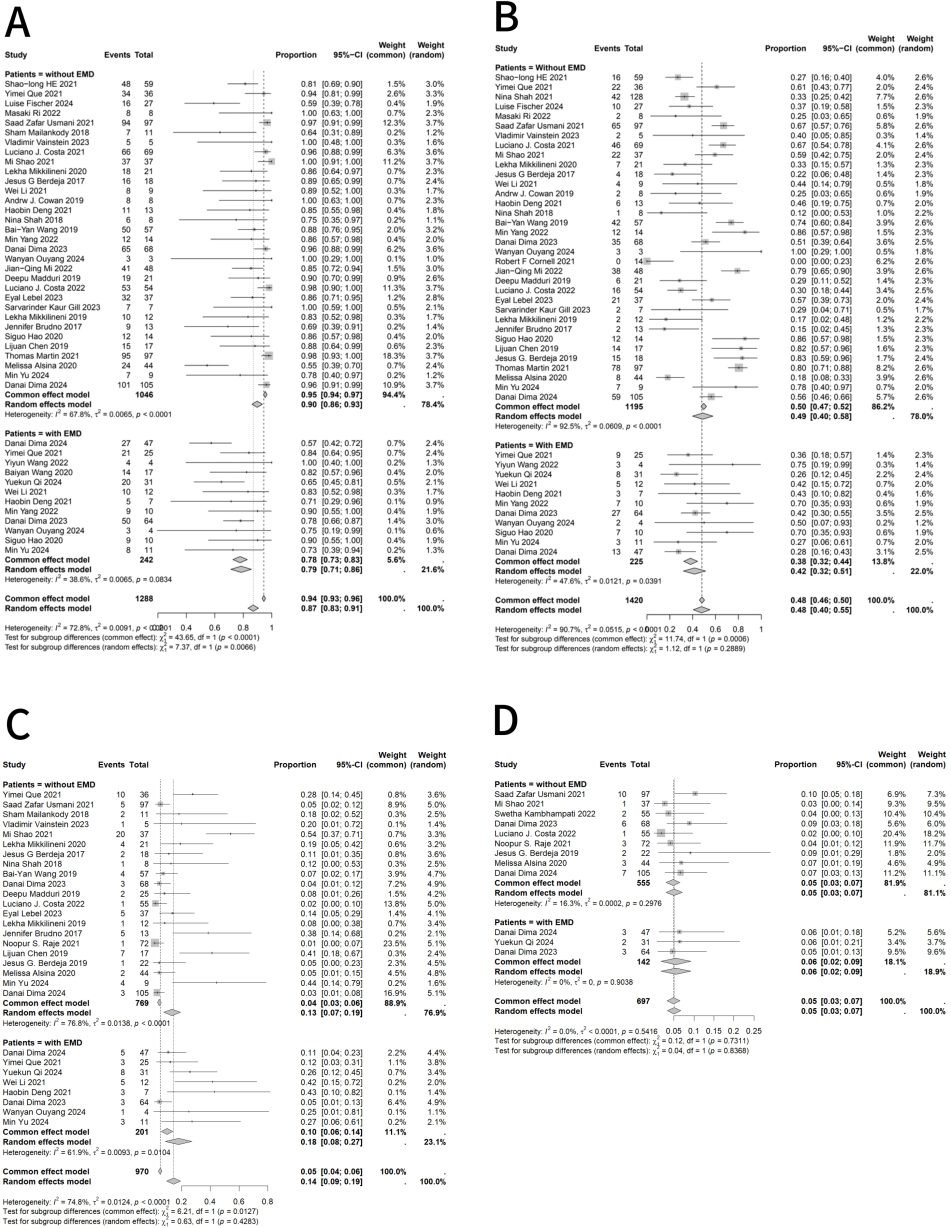
Pooled response rates and grade ≥3 adverse event rates in RRMM patients with and without extramedullary disease following BCMA-directed CAR T therapy. **(A)** Forest plot of the pooled objective response rate (ORR) in RRMM patients with extramedullary disease (EMD) versus those without EMD. **(B)** Forest plot of the pooled complete response (CR) rate in patients with and without EMD. **(C)** Pooled incidence of grade ≥3 cytokine release syndrome (CRS) across the two subgroups. **(D)** Pooled incidence of grade ≥3 immune effector cell–associated neurotoxicity syndrome (ICANS) in patients with and without EMD. Random- or fixed-effects models were applied based on heterogeneity (I²statistics). P value < 0.05 indicates statistical significance, meaning that the observed difference is unlikely to have occurred by chance under the null hypothesis.

A total of 35 studies reported the ORR as a clinical endpoint. A random-effects model was applied to evaluate heterogeneity between RRMM patients with and without EMD. Initial analysis indicated no statistically significant difference between the two groups; therefore, outlier-adjusted data were used for pooling. The combined ORR (**Figure 2A**) was 79% (95%CI: 71%–86%) in the EMD group and 90% (95%CI: 86%–93%) in the non-EMD group, with substantial heterogeneity (I²= 72.8% > 50%) and a statistically significant difference (P= 0.0066 < 0.05). A total of 36 studies reported complete response (CR) as a clinical outcome. Using a random-effects model, the pooled CR rates (**Figure 2B**) were 42% (95%CI: 32%–51%) for RRMM patients with EMD and 49% (95%CI: 40%–58%) for those without EMD. Although heterogeneity was high (I²= 90.7% > 50%), the between-group difference was not statistically significant (P= 0.2889 > 0.05).

#### 3.3.2 Survival

A total of 28 studies reported progression-free survival (PFS) or overall survival (OS) outcomes. However, due to substantial heterogeneity in reporting formats and the absence of individual patient-level time-to-event data, a formal pooled survival analysis could not be performed. Therefore, survival outcomes were summarized descriptively based on the reported data in the included studies, without reconstruction or additional assumptions. Among the studies involving RRMM patients with EMD, the reported median PFS ranged from 3 to 18.8 months, while the reported median OS ranged from 6 to 13.9 months. In comparison, studies involving RRMM patients without EMD showed a broader range, with reported median PFS from 1 to 38 months and median OS from 12.2 to 38 months.

### 3.4 Safety

The primary adverse events associated with CAR T-cell therapy include cytokine release syndrome (CRS) and immune effector cell–associated neurotoxicity syndrome (ICANS). Hematologic toxicities, such as neutropenia and infections, are also commonly reported in CAR T-cell therapy for multiple myeloma; however, these outcomes were not consistently reported across studies and were therefore not included in the pooled analysis. Using a random-effects model to analyze heterogeneity between RRMM patients with and without EMD, 25 studies reported grade ≥3 CRS. The pooled incidence of grade ≥3 CRS (**Figure 2C**) was 18% (95%CI: 8%–27%) in patients with EMD and 13% (95%CI: 7%–19%) in those without, with no statistically significant difference between the two groups (P= 0.4283).

For ICANS, a fixed-effects model was applied. Ten studies reported grade ≥3 ICANS, and the pooled incidence (**Figure 2D**) was 6% (95%CI: 2%–9%) in patients with EMD and 5% (95%CI: 3%–7%) in those without. Heterogeneity was negligible (I²= 0.00%), and the difference between groups was not statistically significant (P= 0.7311).

### 3.5 Sensitivity analysis

**Figure 3:**
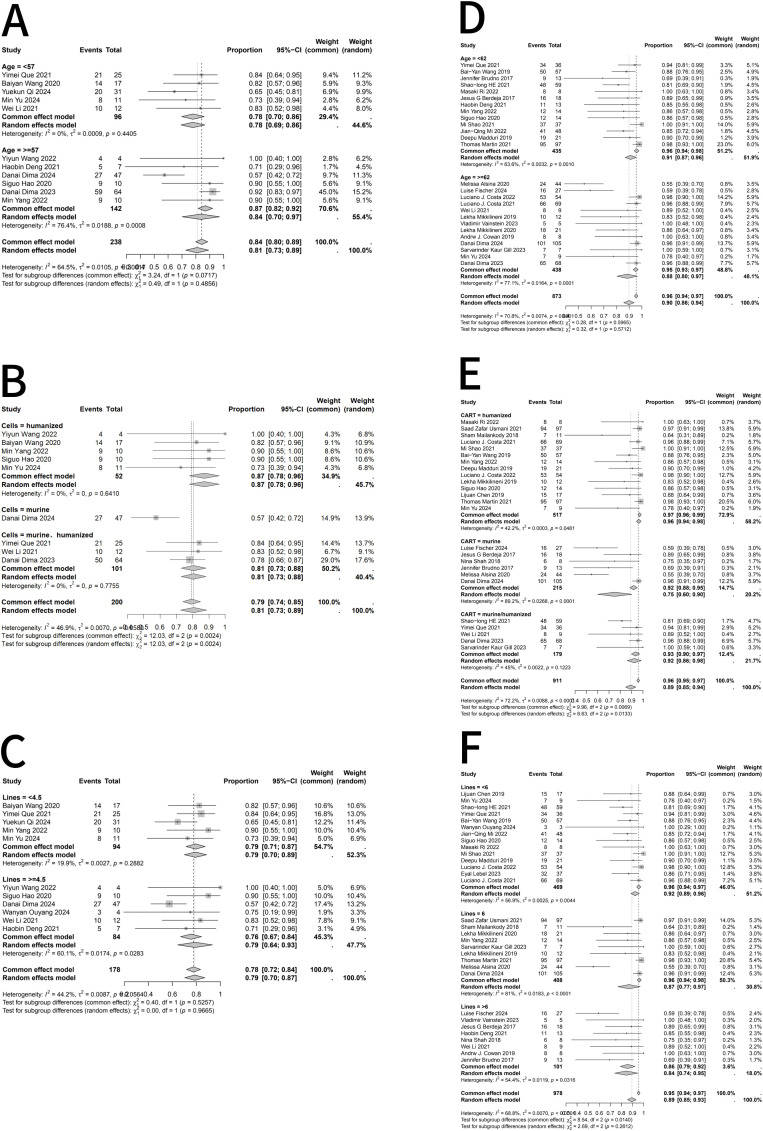
Subgroup analyses of objective response rate (ORR) in RRMM patients with and without extramedullary disease (EMD) following BCMA-directed CAR T-cell therapy. **(A)** Subgroup analysis of ORR by age in RRMM patients with EMD. **(B)** Subgroup analysis of ORR by CAR T-cell construct type in RRMM patients with EMD. **(C)** Subgroup analysis of ORR by number of prior therapy lines in RRMM patients with EMD. **(D)** Subgroup analysis of ORR by age in RRMM patients without EMD. **(E)** Subgroup analysis of ORR by CAR T-cell construct type in RRMM patients without EMD. **(F)** Subgroup analysis of ORR by number of prior therapy lines in RRMM patients without EMD. Fixed- or random-effects models were applied according to heterogeneity (I²statistics). A P value < 0.05 indicates statistical significance, meaning the observed difference is unlikely to have occurred by chance under the null hypothesis.

**Figure 4:**
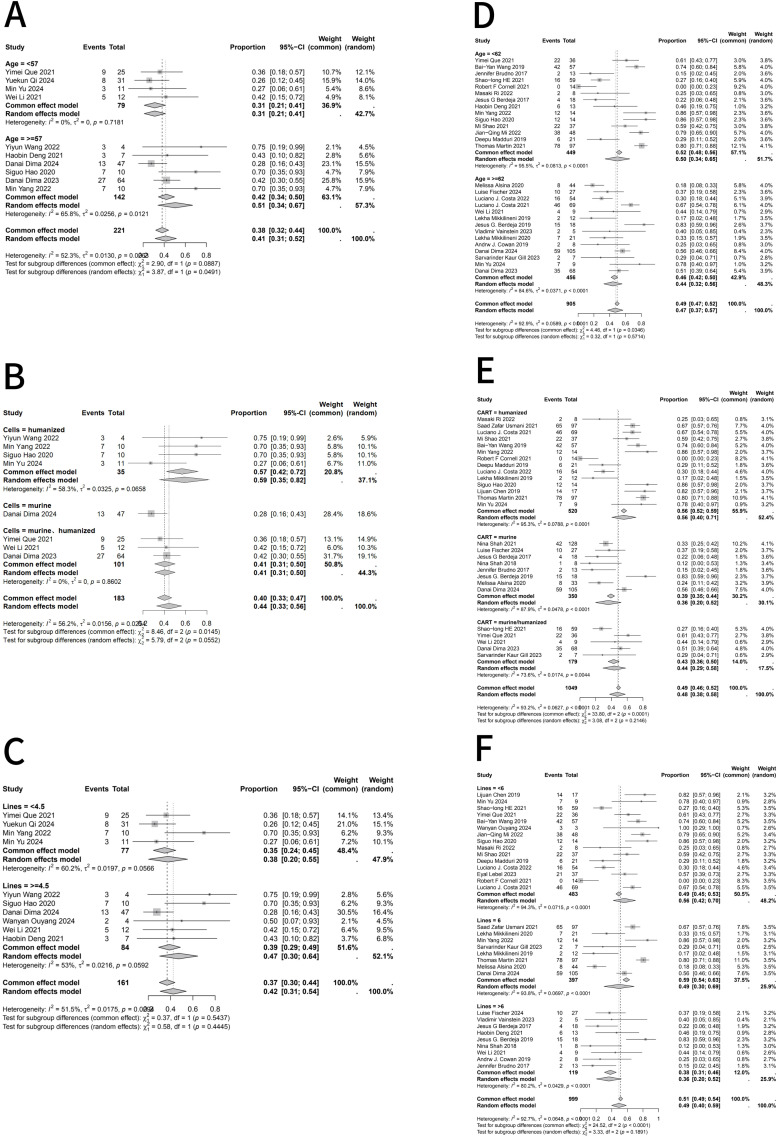
Subgroup analyses of complete response (CR) rates in RRMM patients with and without extramedullary disease following BCMA-directed CAR T therapy. **(A)** Subgroup analysis of CR in RRMM patients with EMD stratified by age. **(B)** Subgroup analysis of CR in RRMM patients with EMD stratified by CAR T-cell construct type (murine vs. humanized). **(C)** Subgroup analysis of CR in RRMM patients with EMD stratified by number of prior treatment lines. **(D)** Subgroup analysis of CR in RRMM patients without EMD stratified by age. **(E)** Subgroup analysis of CR in RRMM patients without EMD stratified by CAR T-cell construct type. **(F)** Subgroup analysis of CR in RRMM patients without EMD stratified by number of prior treatment lines. Random- or fixed-effects models were applied according to heterogeneity (I²statistics). A P value < 0.05 indicates statistical significance, meaning that the observed subgroup difference is unlikely to have occurred by chance under the null hypothesis.

**Figure 5:**
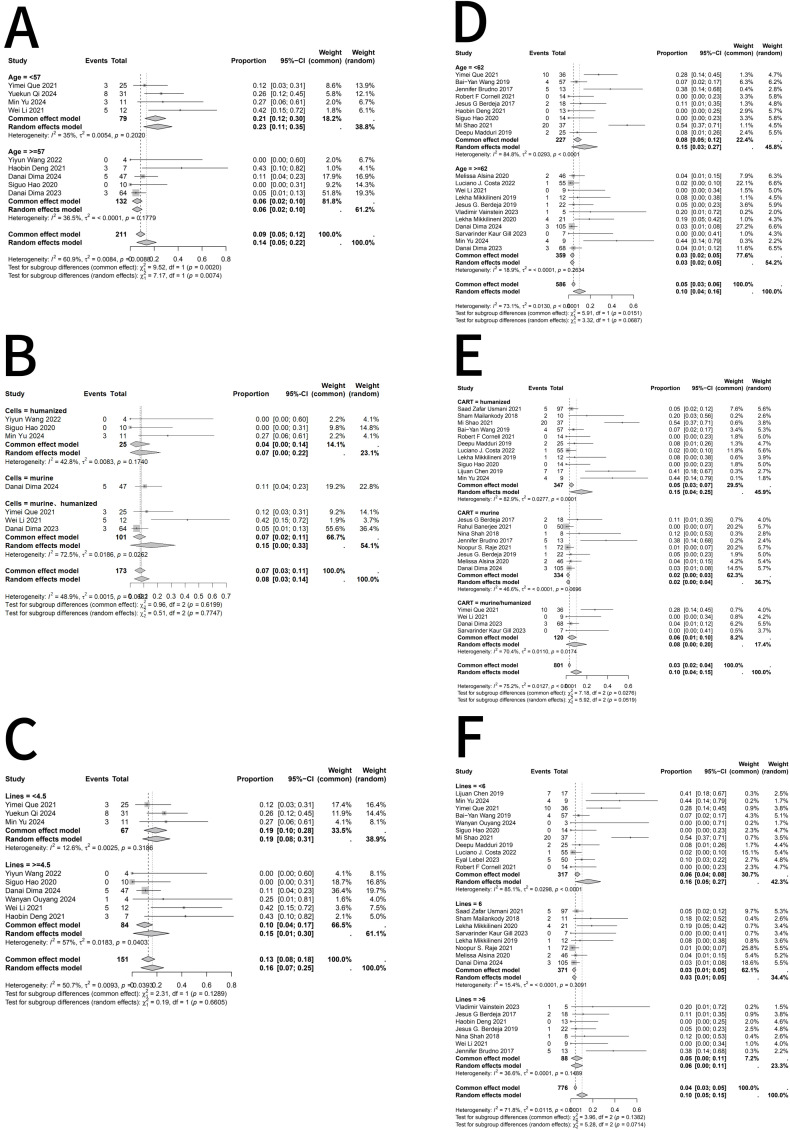
Subgroup analyses of grade ≥3 CRS in RRMM patients with and without extramedullary disease following BCMA-directed CAR T therapy. **(A)** Subgroup analysis of grade ≥3 CRS by age in RRMM patients with extramedullary disease (EMD). **(B)** Subgroup analysis of grade ≥3 CRS by CAR T-cell construct (murine vs. humanized) in patients with EMD. **(C)** Subgroup analysis of grade ≥3 CRS by prior lines of therapy in patients with EMD. **(D)** Subgroup analysis of grade ≥3 CRS by age in RRMM patients without EMD. **(E)** Subgroup analysis of grade ≥3 CRS by CAR T construct type in patients without EMD. **(F)** Subgroup analysis of grade ≥3 CRS by prior lines of therapy in patients without EMD. Random- or fixed-effects models were selected based on subgroup heterogeneity (I²statistics). A P value < 0.05 indicates statistical significance, meaning that the probability of observing such differences under the null hypothesis is less than 5%.

Subgroup analyses were performed because heterogeneity varied across outcomes, with I²values <50% for ICANS but >50% for ORR, CR, and CRS. Considering that patient age, CAR T-cell construct type, and prior lines of therapy might contribute to heterogeneity, Subgroup analyses of ORR (**Figure 3**), CR (**Figure 4**), and CRS (**Figure 5**) were conducted for RRMM patients with and without EMD. When stratifying by age and prior lines of therapy using the median values of the extracted data as the cutoff, the results indicated that in the EMD group, age influenced CR and CRS, while CAR T-cell type affected ORR; prior lines of therapy had no significant impact. In the non-EMD group, only CAR T-cell type was associated with variations in ORR. These findings suggest that the type of CAR T-cell construct is a major source of heterogeneity in objective response rates.

Sensitivity analyses were further conducted for ORR, CR, and CRS in both EMD and non-EMD groups. Except for one study affecting CRS in the EMD group and one study affecting CR in the non-EMD group, all other studies remained within the original confidence intervals, indicating stable results. Although multiple imputation and interquartile range–based outlier detection were applied, only ORR showed a meaningful change after removing outliers—the between-group difference became statistically significant. For other outcomes, these data-processing steps either increased heterogeneity or lowered the pooled effect estimates, suggesting that such adjustments did not improve data consistency and might be unsuitable for these datasets. Therefore, we retained the outlier-adjusted ORR results for further interpretation, while all other outcomes were analyzed using the original datasets due to their overall stability.

### 3.6 Publication bias

Publication bias was assessed using Egger’s test. In the EMD group, no significant publication bias was detected for the pooled CR analysis (P= 0.068), nor for the pooled ORR analysis (P= 0.125). However, significant publication bias was observed for grade ≥3 CRS (P= 0.002). After trim-and-fill adjustment, the P value increased to 0.095, indicating that the initially significant result became non-significant.

In the non-EMD group, no significant publication bias was detected for the pooled CR analysis (P= 0.229). In contrast, significant publication bias was identified for ORR (P< 0.001). Trim-and-fill adjustment did not alter the overall result (P< 0.001), and the result remained statistically significant after adjustment, indicating limited impact of publication bias on the ORR findings. For grade ≥3 CRS, significant publication bias was again observed (P< 0.001). After trim-and-fill correction, the P value increased to 0.043, suggesting a reduction in statistical significance after adjustment and indicating that publication bias may influence CRS estimates.

Overall, while publication bias appears to affect analyses involving grade ≥3 CRS, the remaining outcomes were relatively robust.

## 4 Discussion

### 4.1 Efficacy and safety of CAR T therapy in RRMM patients with EMD

BCMA-targeted chimeric antigen receptor (CAR) T-cell therapy has been regarded as a highly promising modality for patients with relapsed or refractory multiple myeloma (RRMM), with generally manageable toxicity profiles. Previous studies have suggested that although responses can be achieved in patients presenting with extramedullary infiltration and high tumor burden, the durability of these responses has often been limited, indicating that CAR T therapy may confer only partial clinical benefit in this particularly high-risk population (15). In our meta-analysis, an objective response rate of 79% (95%CI: 71%–86%) and a complete response rate of 42% (95%CI: 32%–51%) were observed among RRMM patients with EMD following BCMA-directed CAR T treatment.

Given the inherently aggressive nature of extramedullary multiple myeloma (EMM), characterized by high-risk biology and dismal clinical outcomes, the efficacy of conventional therapies has historically been limited in this subgroup, with reported overall response rates typically below 30–40% and limited durability of response in patients with extramedullary disease. The response rates observed in our analysis therefore suggest that BCMA-targeted CAR T therapy demonstrates substantial clinical activity in this high-risk population, consistent with previously reported outcomes, rather than establishing superiority over conventional therapies. Regarding safety, the pooled incidences of grade ≥3 cytokine release syndrome (CRS) and immune effector cell-associated neurotoxicity syndrome (ICANS) were 18% (95%CI: 8%–27%) and 6% (95%CI: 2%–9%), respectively, indicating that treatment-related toxicities were generally controllable in this high-risk cohort.

Taken together, these findings support the notion that BCMA-directed CAR T therapy offers meaningful clinical activity and an acceptable safety profile for RRMM patients with extramedullary involvement. However, to more comprehensively characterize the durability of response and long-term safety in this population, large-scale studies with extended follow-up are still warranted.

### 4.2 Comparison of the efficacy and safety of CAR T therapy in RRMM patients with and without EMD

Previous meta-analyses have reported that, among patients with RRMM treated with CAR T-cell therapies targeting seven different antigens, the ORR was approximately 77%, the complete response rate was 37%, and the pooled incidences of grade 3–4 cytokine release syndrome (CRS) and neurotoxicity were 14% and 13%, respectively (57). Another meta-analysis evaluating BCMA-directed CAR T therapy in Chinese RRMM patients demonstrated an ORR of 87% (95%CI: 0.84–0.91), a stringent complete response rate of 67% (95%CI: 0.58–0.75), a complete response rate of 57% (95%CI: 0.53–0.61), a grade ≥3 CRS incidence of 16% (95%CI: 0.08–0.26), an ICANS incidence of 11% (95%CI: 0.07–0.16), and a grade ≥3 ICANS rate of 0%. These findings are broadly consistent with those observed in our analysis of RRMM patients with EMD.

When the efficacy of CAR T therapy was compared between RRMM patients with and without extramedullary involvement, pooled analyses revealed objective response rates of 79% (95%CI: 71%–86%) and 90% (95%CI: 86%–93%), respectively. The difference between the two groups was statistically significant (I²= 72.8%, P= 0.0066). The corresponding complete response rates were 42% (95%CI: 32%–51%) and 49% (95%CI: 40%–58%), a difference that did not reach statistical significance (I²= 90.7%, P= 0.2889). These findings suggest that although RRMM patients with EMD tend to exhibit lower objective and complete response rates compared with those without extramedullary involvement, the magnitude of difference in complete response may not be clinically significant. Because most studies reported survival outcomes only as point estimates for PFS and OS, and individual-level time-to-event data were unavailable, survival outcomes could only be summarized descriptively, and a formal pooled survival analysis could not be reliably performed.

Regarding safety, the incidences of grade ≥3 CRS were 18% (95%CI: 8%–27%) in patients with EMD and 13% (95%CI: 7%–19%) in those without (P= 0.4283). For grade ≥3 ICANS, the pooled incidences were 6% (95%CI: 2%–9%) and 5% (95%CI: 3%–7%), respectively, with no significant differences (I²= 0.00%, P= 0.7311). These results indicate that while high-grade CRS and ICANS may occur slightly more frequently in patients with EMD, the differences are not statistically meaningful, and the available evidence is insufficient to conclude that one group experiences superior safety outcomes over the other.

Subgroup analyses suggested a potential associated between CAR T-cell construct type and variability in objective response rates. This observation underscores the possibility that different CAR T-cell designs may exert distinct therapeutic effects, highlighting the need for future investigations to evaluate the performance of specific CAR T constructs in defined clinical subpopulations.

### 4.3 Limitations

Several limitations should be acknowledged in this study. First, considerable heterogeneity was present among the included studies due to variations in patient selection, sample size, CAR T-cell constructs, number of prior therapies, history of hematopoietic stem-cell transplantation, and duration of follow-up. As a result, caution is warranted when interpreting the pooled estimates. Second, discrepancies were noted in the reporting of PFS and OS across studies. Due to the lack of individual patient-level time-to-event data and inconsistent reporting formats, survival outcomes could only be summarized descriptively, precluding a formal time-to-event meta-analysis. Therefore, survival findings should be interpreted with caution. In addition, a formal meta-analysis of survival outcomes (e.g., using hazard ratios) was not feasible due to the absence of consistently reported time-to-event effect measures across studies.

Taken together, these limitations highlight the necessity for future multicenter studies with larger sample sizes and extended follow-up to more reliably evaluate the efficacy and safety of CAR T therapy in RRMM, particularly among patients with EMD. Further research is also required to optimize CAR T therapeutic strategies, reduce treatment-associated toxicity, and enhance the durability and breadth of clinical responses.

## 5 Conclusion

This meta-analysis demonstrated that CAR T-cell therapy represents a promising treatment option for patients with relapsed or refractory MM complicated by EMD. High response rates were observed, and CAR T therapy may offer meaningful clinical benefit. Although treatment-related adverse events such as cytokine release syndrome and immune effector cell–associated neurotoxicity syndrome were reported, these events were generally manageable, indicating an acceptable safety profile. Collectively, these findings suggest that CAR T therapy may offer meaningful clinical benefit for RRMM patients with extramedullary involvement.

Given that EMD is an established adverse prognostic factor for both PFS and OS and is associated with high-risk disease characteristics, lower objective and complete response rates were noted in comparison with patients without extramedullary involvement. However, due to limitations inherent to the included studies—such as small sample sizes, modest effect sizes, and variable study quality—there remains insufficient evidence to definitively determine whether the safety profile differs between patients with and without EMD following CAR T therapy. Larger, high-quality randomized controlled trials with longer follow-uPare therefore warranted to further validate these findings and to optimize CAR T therapeutic strategies in this high-risk population.

## Data Availability

The original contributions presented in the study are included in the article/supplementary material. Further inquiries can be directed to the corresponding authors.
